# Circ_0000527 promotes osteosarcoma cell progression through modulating miR-646/ARL2 axis

**DOI:** 10.18632/aging.202602

**Published:** 2021-02-22

**Authors:** Xiangkun Wu, Lihua Yan, Yongxi Liu, Lilin Shang

**Affiliations:** 1 Department of Orthopaedic Surgery, Nanyang Second People's Hospital, Nanyang 473000, Henan, China; 2 Department of Medical Oncology, Nanyang Second People's Hospital, Nanyang 473000, Henan, China

**Keywords:** osteosarcoma, circ_0000527, ARL2, miR-646

## Abstract

Accumulating evidence shows that circRNAs play critical roles in the development of human tumors. We observed that circ_0000527 was overexpressed in osteosarcoma cells (SAOS-2, HOS, MG-63 and U2OS) compared in hFOB1.19 cells. We demonstrated that the circ_0000527 level was higher in osteosarcoma specimens than in non-tumor specimens. The ectopic expression of circ_0000527 was shown to induce cell growth, cell cycle progression and the secretion of inflammatory mediators, including IL-1β, IL-6, IL-8 and TNF-α. We demonstrated that circ_0000527 sponges miR-646 in osteosarcoma cells and that ARL2 is a target gene of miR-646. MiR-646 expression was decreased and ARL2 was overexpressed in osteosarcoma cells (SAOS-2, HOS, MG-63 and U2OS) compared to hFOB1.19 cells. Overexpression of circ_0000527 was demonstrated to induce ARL2 expression in MG-63 cells. We showed that miR-646 was downregulated in osteosarcoma specimens compared to that of non-tumor specimens and that the level of circ_0000527 was negatively correlated with miR-646 expression in osteosarcoma specimens. The elevated expression of circ_0000527 was shown to promote cell growth and cell cycle progression by modulating miR-646 expression. The ectopic expression of circ_0000527 was shown to promote cell growth, cell cycle progression and the secretion of inflammatory mediators by modulating ARL2. The present study suggested that the circ_0000527/miR-646/ARL2 axis may be a potential treatment target for osteosarcoma.

## INTRODUCTION

Osteosarcoma is the most frequent type of primary malignant bone tumor in adolescents and children [1–5]. The predicted worldwide incidence is four million cases every year, with a high incidence at 15–19 years of age [6–9]. Osteosarcoma is locally destructive with a high rate of metastasis to other organs, especially to the lung [10–12]. Despite the rapid advances in treatment strategies, including adjuvant chemotherapy, radiotherapy and wide local tumor excision, the 5-year survival rate remains unsatisfactory [13–16]. Thus, it is urgent to study the fundamental mechanisms and find novel diagnostic and therapeutic targets for osteosarcoma.

Accumulating evidence has shown that circRNAs can modulate gene expression by acting as competing endogenous RNAs (ceRNAs) for miRNAs and their target genes [17–21]. CircRNAs are deregulated in several tumors, such as nasopharyngeal carcinoma, retinoblastoma, glioma, cervical cancer and osteosarcoma [22–26]. CircRNAs play critical roles in many cell functions, including metabolism, development, differentiation, invasion and apoptosis [27–29]. Recently, Zhang et al. [30] have illustrated that circ_0000527 is upregulated in retinoblastoma cells and tissues, inhibits cell apoptosis and induces cell invasion, growth and migration. However, its function in osteosarcoma remains elusive.

We demonstrated that the circ_0000527 level is higher in osteosarcoma specimens than in non-tumor specimens. We showed that the ectopic expression of circ_0000527 induces cell growth, cell cycle progression, invasion and secretion of inflammatory mediators, including IL-1β, IL-6, IL-8 and TNF-α.

## RESULTS

### circ_0000527, miR-646 and ARL2 expression in osteosarcoma cells

**Figure 1:**
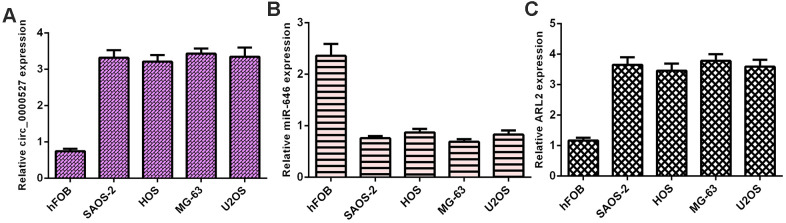
**circ_0000527, miR-646 and ARL2 expression in osteosarcoma cells.** (**A**) The expression of circ_0000527 in osteosarcoma cells (SAOS-2, HOS, MG-63 and U2OS) and hFOB1.19 was determined by qRT-PCR assay. (**B**) miR-646 was decreased in osteosarcoma cells (SAOS-2, HOS, MG-63 and U2OS) compared to hFOB1.19 cells. (**C**) The expression of ARL2 in osteosarcoma cells (SAOS-2, HOS, MG-63 and U2OS) and hFOB1.19 was measured using qRT-PCR assay.

We observed that circ_0000527 was overexpressed in osteosarcoma cells (SAOS-2, HOS, MG-63 and U2OS) compared to hFOB1.19 cells (Figure 1A). MiR-646 expression was decreased in osteosarcoma cells (SAOS-2, HOS, MG-63 and U2OS) compared to hFOB1.19 cells (Figure 1B). ARL2 was overexpressed in osteosarcoma cells (SAOS-2, HOS, MG-63 and U2OS) compared to hFOB1.19 cells (Figure 1C).

### Circ_0000527 expression is higher in osteosarcoma specimens

**Figure 2:**
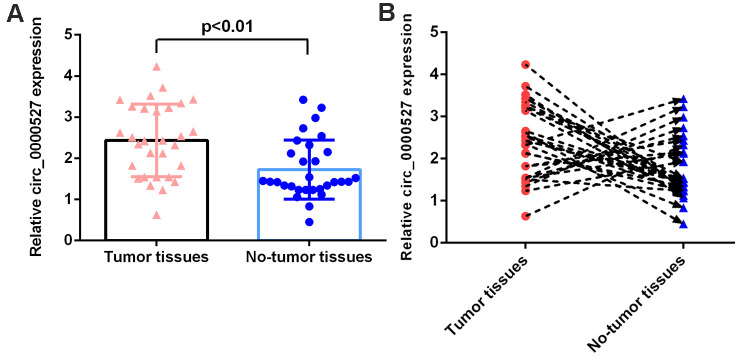
**circ_0000527 expression in osteosarcoma specimens.** (**A**) The level of circ_0000527 in osteosarcoma specimens and non-tumor specimens was measured by a qRT-PCR assay. (**B**) The level of circ_0000527 was upregulated in 20 cases (20/30, 66.7%) compared to non-tumor specimens.

Then, we determined the expression of circ_0000527 in the osteosarcoma specimens. We observed that the circ_0000527 level was higher in the osteosarcoma specimens than in the non-tumor specimens (Figure 2A).

The level of circ_0000527 was upregulated in 20 cases (20/30, 66.7%) compared to the non-tumor specimens (Figure 2B).

### Ectopic expression of circ_0000527 induces cell growth and cell cycle progression

**Figure 3:**
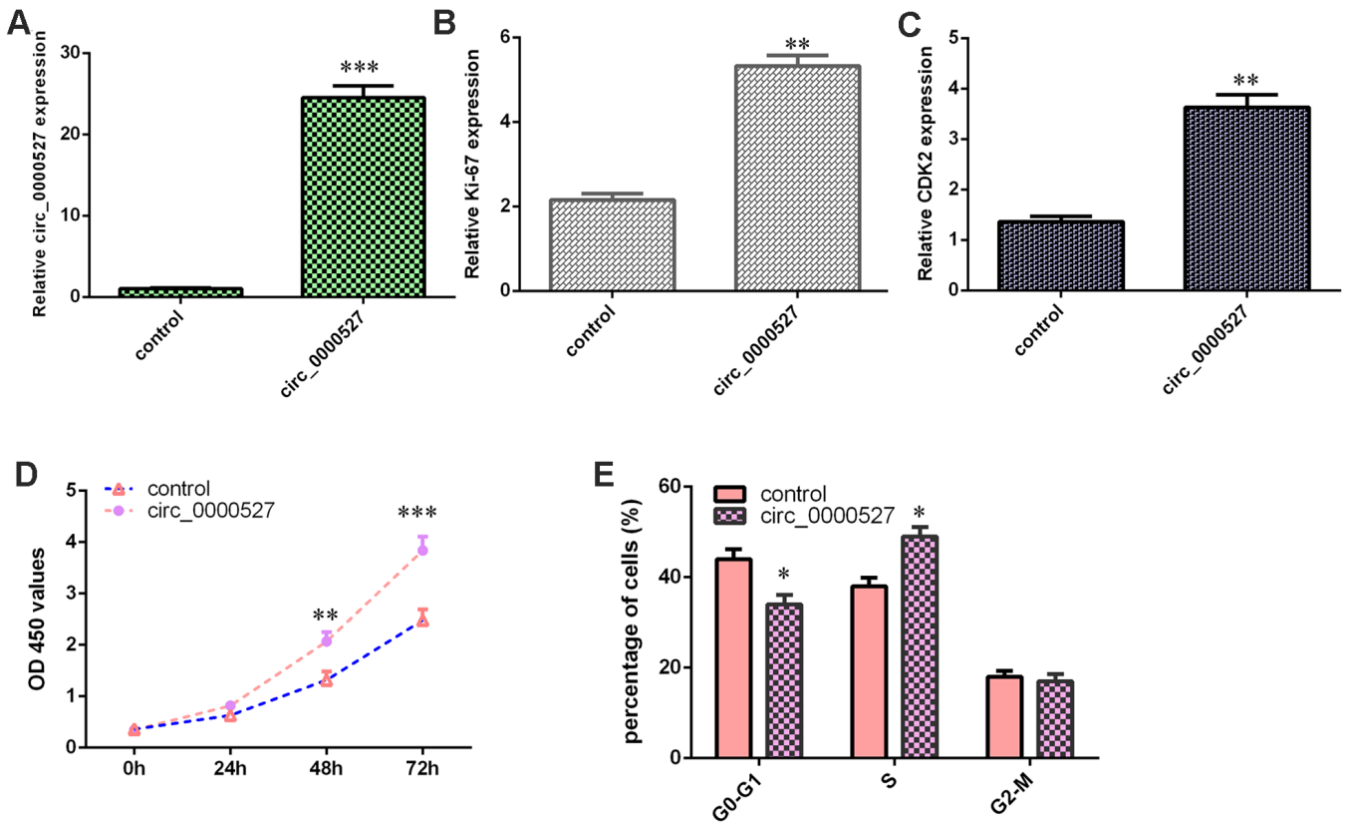
**Ectopic expression of circ_0000527 induced cell growth, cell cycle progression and invasion.** (**A**) The level of circ_0000527 was determined by a qRT-PCR assay. (**B**) The elevated expression of circ_0000527 enhanced ki-67 expression in MG-63 cells. (**C**) The level of CDK2 was determined using qRT-PCR analysis. (**D**) The ectopic expression of circ_0000527 induced cell growth in MG-63 cells. (**E**) The overexpression of circ_0000527 induced cell cycle progression in MG-63 cells. *p<0.05, ** p<0.01, ***p<0.001.

The level of circ_0000527 was upregulated in MG-63 cells after treatment with pcDNA-circ_0000527 compared to that of the control group (Figure 3A). The elevated expression of circ_0000527 enhanced the expression of ki-67 (Figure 3B) and CDK2 (Figure 3C) in MG-63 cells. The ectopic expression of circ_0000527 induced cell growth in MG-63 cells (Figure 3D). The overexpression of circ_0000527 induced cell cycle progression in MG-63 cells (Figure 3E).

### Overexpression of circ_0000527 promotes the secretion of inflammatory mediators

**Figure 4:**
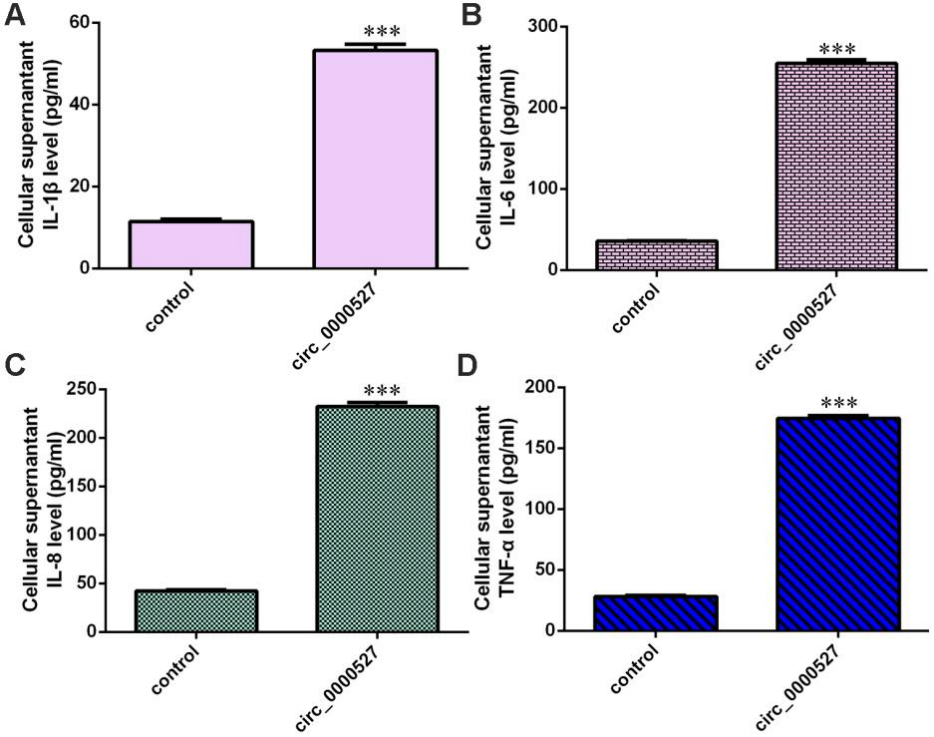
**Overexpression of circ_0000527 promoted secretion of inflammatory mediators.** (**A**) The elevated expression of circ_0000527 induced IL-1β expression in MG-63 cells. (**B**) The expression of IL-6 was determined using an ELISA assay. (**C**) The ectopic expression of circ_0000527 enhanced IL-8 expression. (**D**) The expression of TNF-α was determined by ELISA assay. ***p<0.001.

The elevated expression of circ_0000527 induced IL-1β expression in MG-63 cells (Figure 4A). The overexpression of circ_0000527 promoted IL-6 expression in MG-63 cells (Figure 4B). The ectopic expression of circ_0000527 enhanced the expression of IL-8 (Figure 4C) and TNF-α (Figure 4D) in MG-63 cells.

### Circ_0000527 sponges miR-646 in osteosarcoma cells

**Figure 5:**
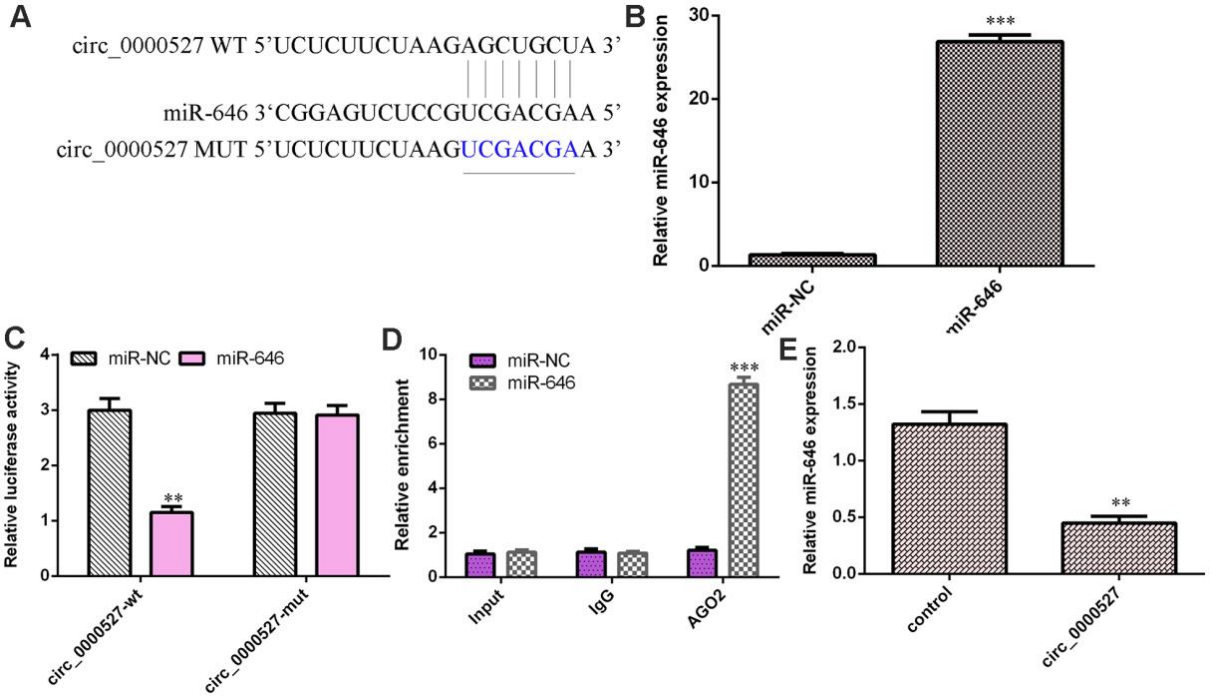
**circ_0000527 sponged miR-646 expression in osteosarcoma cells.** (**A**) As predicted by Starbase, circ_0000527 might be regulated by miR-646. (**B**) The level of miR-646 was measured by a qRT-PCR assay. (**C**) The overexpression of miR-646 significantly decreased the luciferase value of WT circ_0000527 but did not have an effect on mut circ_0000527. (**D**) A RIP assay illustrated that overexpression of miR-646 could be enriched with circ_0000527. (**E**) The elevated expression of circ_0000527 suppressed miR-646 expression in MG-63 cells. ** p<0.01.

As predicted by Starbase, circ_0000527 might be regulated by miR-646 (Figure 5A). The level of miR-646 was upregulated in MG-63 cells after treatment with the miR-646 mimic compared to the control group (Figure 5B). We observed that overexpression of miR-646 significantly decreased the luciferase value of WT circ_0000527 but did not have an effect on mut circ_0000527 (Figure 5C). The RIP assay illustrated that expression of miR-646 could be enriched with circ_0000527 (Figure 5D). The elevated expression of circ_0000527 suppressed miR-646 expression in MG-63 cells (Figure 5E).

### ARL2 is a target gene of miR-646

**Figure 6:**
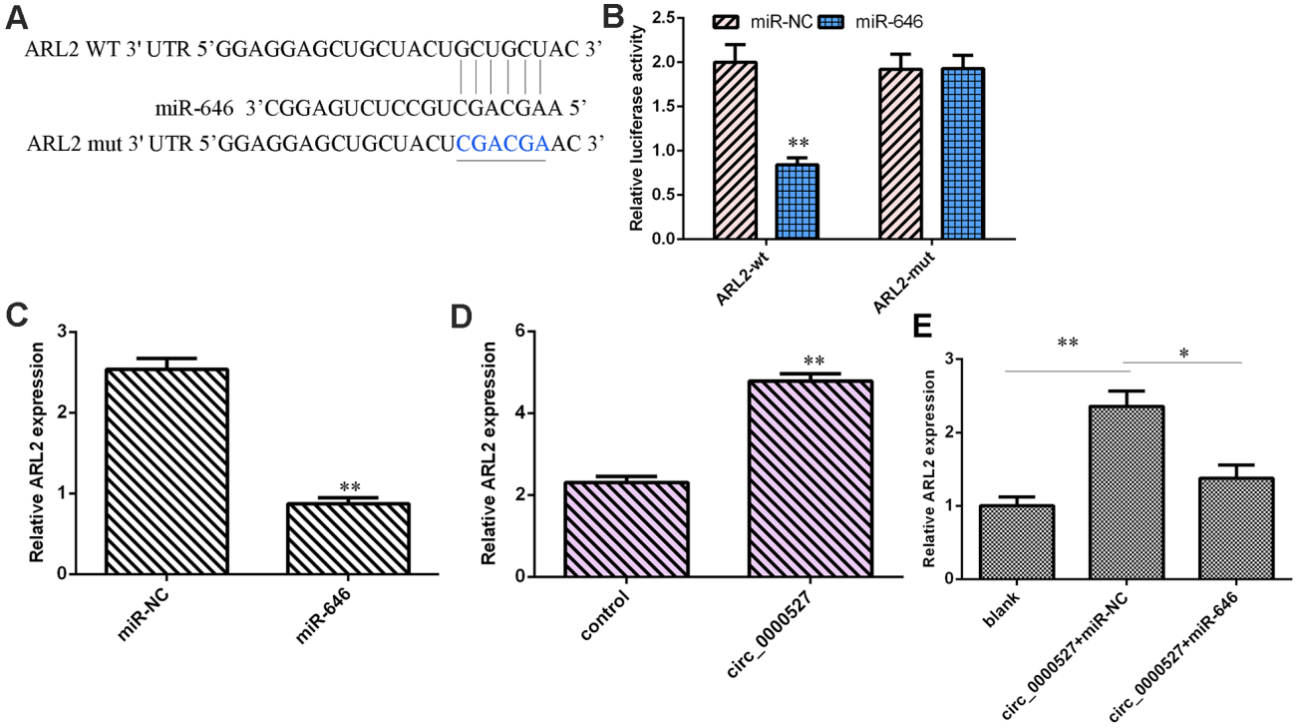
**ARL2 was a target gene of miR-646.** (**A**) As predicted by Targetscan, ARL2 might be regulated by miR-646. (**B**) The overexpression of miR-646 significantly decreased luciferase value of WT ARL2 but did not have an effect on mut ARL2. (**C**) The elevated expression of miR-646 suppressed ARL2 expression in MG-63 cells. (**D**) The expression of ARL2 was measured using a qRT-PCR assay. (**E**) The expression of ARL2 was assessed by qRT-PCR analysis. ** p<0.01.

As predicted by Targetscan, ARL2 expression might be regulated by miR-646 (Figure 6A). We observed that overexpression of miR-646 significantly decreased the luciferase value of WT ARL2 but did not have an effect on mut ARL2 (Figure 6B). The elevated expression of miR-646 suppressed ARL2 expression in MG-63 cells (Figure 6C). The overexpression of circ_0000527 induced ARL2 expression in MG-63 cells (Figure 6D). The ectopic expression of circ_0000527 promoted ARL2 expression, but the overexpression of miR-646 could decrease its expression (Figure 6E).

### MiR-646 expression is downregulated in osteosarcoma

**Figure 7:**
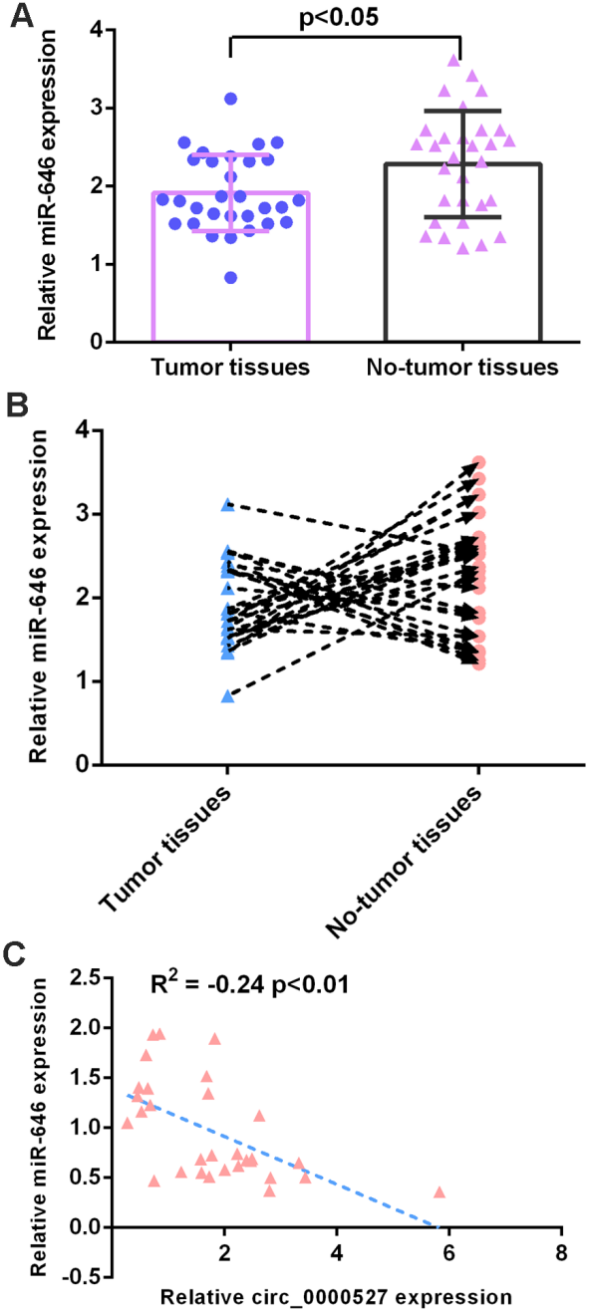
**miR-646 expression was downregulated in osteosarcoma.** (**A**) miR-646 was downregulated in osteosarcoma specimens compared to non-tumor specimens. The expression of miR-646 was detected by a qRT-PCR assay. (**B**) The level of miR-646 was upregulated in 17 cases (17/30, 56.7%) compared to non-tumor specimens. (**C**) The level of circ_0000527 was negatively correlated with miR-646 expression in osteosarcoma specimens.

Furthermore, we demonstrated that miR-646 was downregulated in osteosarcoma specimens compared to non-tumor specimens (Figure 7A). The level of miR-646 was upregulated in 17 cases (17/30, 56.7%) compared to non-tumor specimens (Figure 7B). The level of circ_0000527 was negatively correlated with miR-646 expression in osteosarcoma specimens (Figure 7C).

### Ectopic expression of circ_0000527 promotes cell growth and cell cycle progression by modulating miR-646

**Figure 8:**
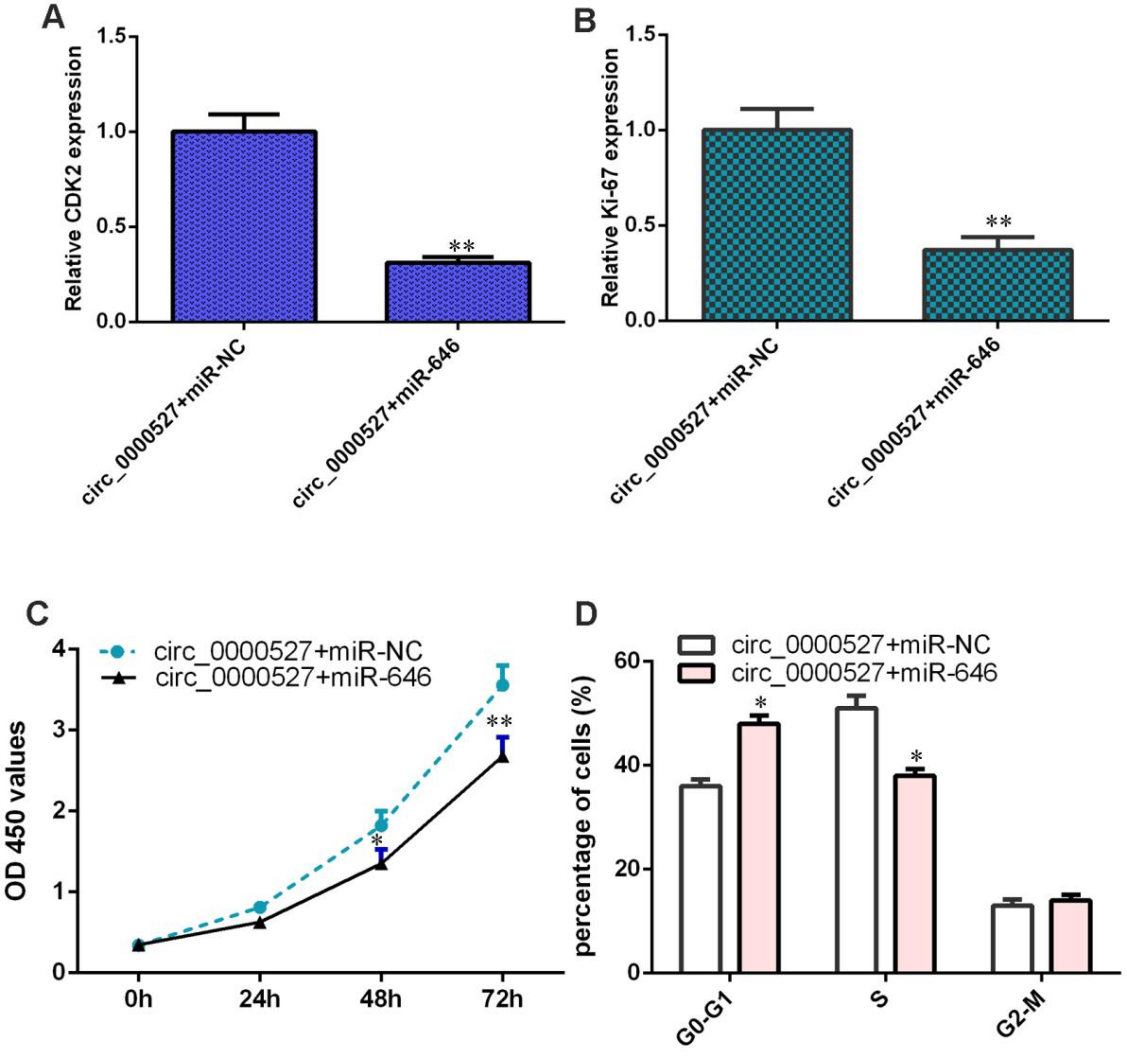
**Ectopic expression of circ_0000527 promoted cell growth and cycle via modulating miR-646.** (**A**) The level of CDK2 was measured by a qRT-PCR assay. (**B**) The expression of ki-67 was determined using a qRT-PCR assay. (**C**) Cell growth was assessed by CCK-8 analysis. (**D**) Cell cycle progression was determined by flow cytometry. *p<0.05, ** p<0.01, ***p<0.001.

The elevated expression of miR-646 decreased expression of CDK2 (Figure 8A) and ki-67 (Figure 8B) in the circ_0000527-overexpressing MG-63 cells. Overexpression of miR-646 suppressed cell proliferation (Figure 8C) and cell cycle progression (Figure 8D) in the circ_0000527-overexpressing MG-63 cells.

### Ectopic expression of circ_0000527 promotes cell growth and cell cycle progression by modulating ARL2

**Figure 9:**
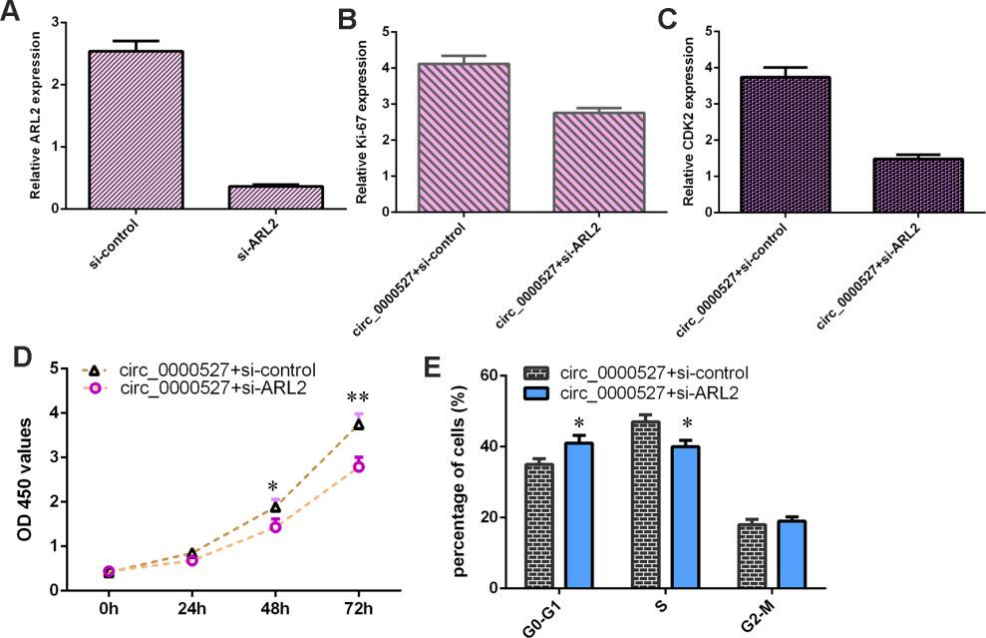
**Ectopic expression of circ_0000527 promoted cell growth, cycle and invasion via modulating ARL2.** (**A**) The expression of ARL2 was determined using qRT-PCR assay. (**B**) The expression of ki-67 was detected using qRT-PCR analysis. (**C**) The level of CDK2 was measured by qRT-PCR assay. (**D**) Inhibition expression of ARL2 suppressed cell proliferation in circ_0000527-overexpressing MG-63 cell. (**E**) Cell cycle was determined by flow cytometry. *p<0.05,** p<0.01, ***p<0.001.

The level of ARL2 was downregulated in MG-63 cells after treatment with ARL2 siRNA compared to that of the control group (Figure 9A). Knockdown of ARL2 decreased the expression of ki-67 (Figure 9B) and CDK2 (Figure 9C) in the circ_0000527-overexpressing MG-63 cells. Inhibition of ARL2 suppressed cell proliferation (Figure 9D) and cell cycle progression (Figure 9E) in the circ_0000527-overexpressing MG-63 cells.

### Elevated expression of circ_0000527 promotes secretion of inflammatory mediators by regulating ARL2

**Figure 10:**
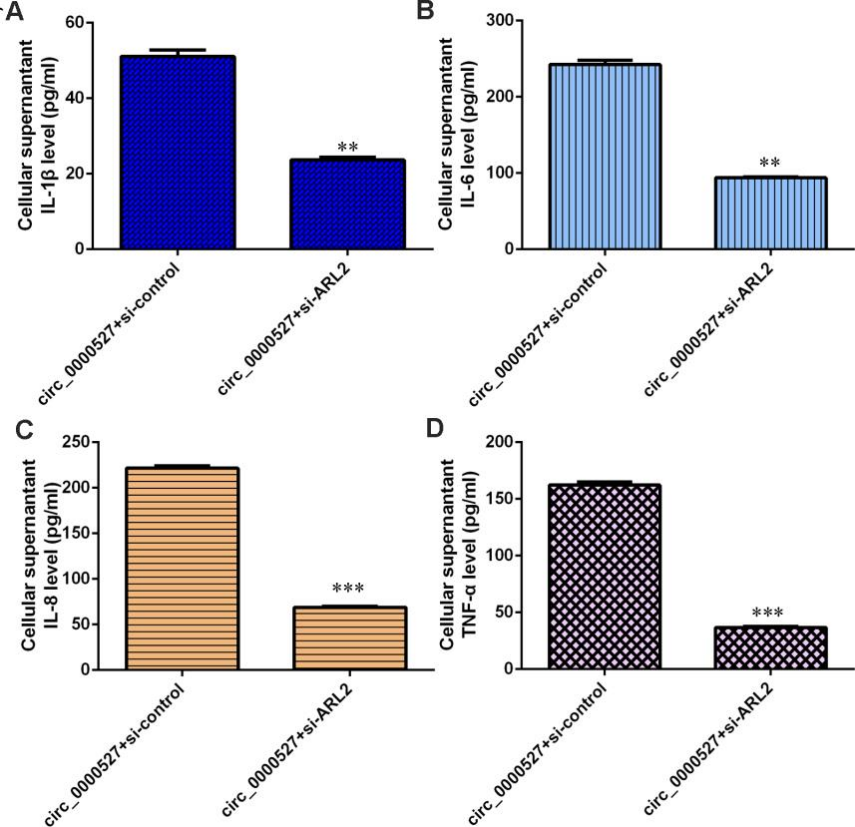
**Elevated expression of circ_0000527 promoted secretion of inflammatory mediators through regulating ARL2.** (**A**) The knockdown of ARL2 suppressed IL-1β expression in circ_0000527-overexpressing MG-63 cells. (**B**) The expression of IL-6 was assessed by ELISA. (**C**) The expression of IL-8 was assessed by ELISA. (**D**) The expression of TNF-α was assessed by ELISA. ** p<0.01, ***p<0.001.

Knockdown of ARL2 suppressed IL-1β expression (Figure 10A) and IL-6 expression (Figure 10B) in the circ_0000527-overexpressing MG-63 cells. Inhibition of ARL2 inhibited expression of IL-8 expression (Figure 10C) and TNF-α (Figure 10D) in the circ_0000527-overexpressing MG-63 cells.

## DISCUSSION

Increasing evidence has shown that circRNAs play critical roles in the development of human tumors [31–33]. Existing studies illustrate that circRNAs can modulate multidrug resistance, metastasis and growth in human malignancies [34–37]. For instance, Ding et al. [38] illustrated that circ_0005909 was overexpressed in osteosarcoma cells and specimens and was correlated with a poor survival rate. The inhibition of circ_0005909 has been shown to suppress cell viability, invasion, EMT and migration *in vitro* and to decrease cancer growth *in vivo* partially by regulating miR-936/HMGB1. Lin et al. [39] demonstrated that circEIF4G2 was overexpressed in osteosarcoma cells and specimens and that the knockdown of circEIF4G2 inhibited osteosarcoma cell invasion, growth and migration through modulating miR-218. Li and colleague illustrated that circ_0003732 was overexpressed in specimens and that overexpression of circ_0003732 induced cell proliferation by regulating the CCNA2/miR-545 axis [40]. Wu et al. [41] indicated that circUBAP2 was overexpressed in osteosarcoma cells and specimens and that knockdown of circUBAP2 inhibited cellular EMT, invasion and growth through modulating the miR-641/ YAP1 axis. Xu et al. [42] illustrated that circTUBGCP3 was overexpressed in osteosarcoma specimens and that overexpression of circTUBGCP3 induced cell migration, survivability and proliferation through sponging miR-30b. Recently, Zhang et al. [30] illustrated that circ_0000527 was upregulated in retinoblastoma cells and tissues and that circ_0000527 overexpression inhibited cell apoptosis and induced cell invasion, growth and migration. Furthermore, Chen et al. [43] also observed that ectopic expression of circ_0000527 enhanced retinoblastoma cell migration, viability and invasion. Exposure to inflammatory cytokines is the principal reason for tumorigenesis, and the regulation of inflammation is an important method for preventing cancer progression [44]. We observed that circ_0000527 is overexpressed in osteosarcoma cells (SAOS-2, HOS, MG-63 and U2OS) compared to hFOB1.19 cells. We demonstrated that the circ_0000527 level is higher in the osteosarcoma specimens than in the non-tumor specimens. We showed that the ectopic expression of circ_0000527 induces cell growth, cell cycle progression, invasion and secretion of inflammatory mediators, including IL-1β, IL-6, IL-8 and TNF-α.

Previously published studies have shown that circRNAs can act as ceRNAs of miRNAs to decrease the activity of miRNAs and modulate gene expression, transcription and binding with these proteins [45, 46]. For example, Zhang et al. [47] noted that circ_0136666 overexpression promoted osteosarcoma tumorigenesis by regulating the ZEB2/miR-593-3p pathway. Fang et al. [48] showed that circ_0000337 was upregulated in osteosarcoma specimens and that the knockdown of circ_0000337 inhibited osteosarcoma cell migration and growth by modulating miR-4458/BACH1. Zhang et al. [49] noted that circ_0002052 induced osteosarcoma progression through regulating the miR-382/STX6 axis. A previous study showed that overexpression of circ_0000527 enhanced retinoblastoma cell migration, viability and invasion by modulating miR-646 [30]. We demonstrated that circ_0000527 sponges miR-646 expression in osteosarcoma cells and that ARL2 is a target gene of miR-646. It was demonstrated that miR-646 decreases and that ARL2 is overexpressed in osteosarcoma cells (SAOS-2, HOS, MG-63 and U2OS) compared to hFOB1.19 cells. Furthermore, we showed that miR-646 is downregulated in osteosarcoma specimens compared to non-tumor specimens. We demonstrated that the level of circ_0000527 is negatively correlated with miR-646 expression in the osteosarcoma specimens. We also showed that the ectopic expression of circ_0000527 promotes cell growth and cell cycle progression by modulating miR-646 expression and that the overexpression of circ_0000527 induces ARL2 expression in MG-63 cells. A previous study showed that microRNA-497-5p suppresses the tumor cell growth of osteosarcoma by targeting ADP ribosylation factor-like protein 2. We showed that the ectopic expression of circ_0000527 promotes cell growth, cell cycle progression, invasion and secretion of inflammatory mediators by modulating ARL2 expression.

In summary, this study revealed that circ_0000527 is overexpressed in osteosarcoma cells and specimens and that ectopic expression of circ_0000527 promotes cell growth, cell cycle progression, invasion and the secretion of inflammatory mediators by modulating ARL2. Therefore, the circ_0000527/miR-646/ARL2 axis may be a potential treatment target for osteosarcoma.

## MATERIALS AND METHODS

### Cell culture, transfection and clinical samples

Osteosarcoma cells (SAOS-2, HOS, MG-63 and U2OS) and hFOB1.19 cells were acquired from the American type culture collection (ATCC) and cultured in DMEM (Invitrogen Inc., USA) supplemented with streptomycin, penicillin and FBS. miR-646, pcDNA circ_0000527 and their controls were synthesized at Shanghai GenePharma. Cell transfection was performed with Lipofectamine 3000 (Invitrogen Inc., USA). Osteosarcoma surgical specimens and control specimens were obtained from Nanyang First People’s Hospital following the criteria approved by the institutional review board. Informed consent was gained from each patient.

### qRT-PCR

Total RNA from cells cultured from the osteosarcoma specimens was collected using a TRIzol kit (Invitrogen Inc, USA). The relative expression of circ_0000527, miR-646 and ARL2 was detected by a qRT-PCR assay using a SYBR Green kit on a Bio-Rad CFX96 PCR system (VisonBio Scientific). U6 snRNA and GAPDH were used as the controls for the miRNA and mRNA, respectively. The method of 2^-DDCt^ was utilized for analysis of the PCR results.

### CCK-8 assay, cell cycle analysis and ELISA

The cells were cultured in 96-well dishes for 0, 24, 48 and 72 hours. At the desired time point, 10 ul of CCK-8 solution from the kit was added into each well. After 4 hours, the OD at 450 nM was assessed. Cytokines in the supernatant were assessed by ELISA. IL-1β, IL-6, IL-8 and TNF-α kits (R&D Systems, USA) were used to determine the IL-1β, IL-6, IL-8 and TNF-α concentrations, respectively. For the cell cycle analysis, the cells were stained with propidium iodide (PI) for half an hour. The cell cycle was assessed by flow cytometry.

### RIP assay

The Magna RIP RBP Immunoprecipitation Kit (Millipore) was used for the RIP assay. The cells were resuspended in the lysis buffer containing RNase and protease inhibitors (Epicentre). The cell lysate was then incubated with beads coated with an AGO2 antibody or a control IgG. After incubation in proteinase K, the immunoprecipitated RNA was isolated and assessed by qRT-PCR.

### Dual-luciferase reporter gene assay

The wt and mut constructs of circ_0000527 and the ARL2 gene were amplified with RT-PCR. The cells were cultured in 24-well dishes and co-transfected with the miR-646 mimic or miR-NC and pGL3-circ_0000527, ARL2-3’UTR-WT, pGL3-circ_0000527 or ARL2-3’UTR-MUT and pRL-TK by Lipofectamine 3000 (Invitrogen Inc, USA). After 48 hours, the firefly and Renilla luciferase values were detected using a dual-luciferase kit (Promega, USA). Renilla luciferase was used as the control.

### Statistical analysis

All results are shown as the mean ± standard deviation. The significant difference between two groups was determined with a Student’s t test. P <0.05 was set as statistically significant.
